# A Driver Screening Method Based on Perception Ability Test of Dangerous Omen

**DOI:** 10.3390/s26051447

**Published:** 2026-02-26

**Authors:** Longfei Chen, Xiaoyuan Wang, Jingheng Wang, Han Zhang, Chenyang Jiao, Bin Wang, Kai Feng, Cheng Shen, Quanzheng Wang, Junyan Han, Tinglin Chen, Zhenwei Lv

**Affiliations:** 1 College of Electromechanical Engineering, Qingdao University of Science and Technology, Qingdao 266000, China; chenlongfei@mails.qust.edu.cn (L.C.); zhanghan@mails.qust.edu.cn (H.Z.); jiaochenyang@mails.qust.edu.cn (C.J.); wangbin@mails.qust.edu.cn (B.W.); fengkai@mails.qust.edu.cn (K.F.); b024030003@mails.qust.edu.cn (C.S.); 0020030005@mails.qust.edu.cn (Q.W.); hanjunyan@mails.qust.edu.cn (J.H.); chentinglin@mails.qust.edu.cn (T.C.); 4023031020@mails.qust.edu.cn (Z.L.); 2 Department of Mathematics, Ohio State University, Columbus, OH 43220, USA

**Keywords:** dangerous omen perception, driver, vehicle, autonomous driving

## Abstract

According to in-depth research on the perception ability of dangerous omens of excellent drivers, references can be provided for the development of brain-like intelligence and its transplantation, as well as applications in the field of autonomous driving, which will improve the active safety and intelligence level of vehicles. Previous studies have shown that there is indeed a dangerous omen before an accident occurs. However, current studies are still unclear about the bio-psychophysiological characteristics exhibited by drivers with high levels of sensory agility when they anticipate potential warning signs, and there is no method for screening such drivers who can perceive dangerous omens proposed by any research. To address the above issues, this paper conducts in-depth research. Firstly, through designing dangerous scenarios and conducting hazard perception tests, we collect physiological, psychological, and physical data, such as drivers’ bioelectrical signals (electroencephalogram and electrocardiogram) and eye movements. Secondly, through playing back experimental videos, actively questioning drivers, and analyzing local changes in their electroencephalogram data, the driver’s ability to identify a dangerous omen and the moment of perception are determined. Thirdly, based on techniques such as the Kolmogorov–Smirnov test and the Mann–Whitney U test, the differences in bioelectrical and eye movement characteristics between drivers who can perceive a dangerous omen and others can be further revealed. Finally, the driver’s bioelectrical and eye movement characteristics are used as latent variables, and their corresponding data are utilized as observation indicators. We construct a structural equation model for screening drivers capable of perceiving a dangerous omen and conduct calibration and validation. This study provides inspirational ideas for empowering vehicles to identify potential hazards, advancing end-to-end and other higher-level autonomous driving technologies, and further enhancing road traffic safety.

## 1. Introduction

If drivers can perceive danger and respond in time before an accident occurs, the probability of such an accident will be significantly reduced. Some drivers possess exceptional sensory abilities, which means that they can not only perceive impending dangers but also anticipate and recognize potential hazards. In other words, they have the ability to perceive dangerous omens. Danger perception is the main focus of current research, namely, perception of dangerous situation. Currently, potential risks or hidden dangers have also been addressed in a few studies on danger perception. These studies indicate that traffic accidents are predictable. There are indeed warning signs before danger occurs, that is, the dangerous omen is objectively present. However, current research is still unclear about the bio-psychological characteristics of drivers who can perceive dangerous omens. No research has proposed a screening method for such drivers. Furthermore, no research has explored in-depth the subjective perception of danger scenarios by these drivers (i.e., the driving scenario from their first-person perspective when they perceive dangerous omens) and analyzed their driving behaviors in such scenarios. In this paper, preliminary exploratory research is conducted to address the aforementioned issues. The bio-psycho-physical characteristics of drivers capable of perceiving hazards are explored. An exploratory driver screening method based on hazardous omen perception tests is presented in this paper to provide inspired insights that could eventually contribute to enhancing vehicle active safety and brain-like intelligence. The main research findings are as follows. We design dangerous scenarios and conduct hazard perception tests to collect physiological, psychological, and physical data, such as drivers’ bioelectrical characteristics and eye movements. By playing back experimental videos, actively questioning drivers, and analyzing local changes in their electroencephalogram (EEG) data, it is determined whether drivers could perceive dangerous situations and when they perceive them. Based on methods such as the Kolmogorov–Smirnov (K-S) test and the Mann–Whitney U (MWU) test, the differences in bioelectrical and oculomotor characteristics between drivers capable of perceiving dangerous omens and others are further revealed. We construct a structural equation model to screen drivers who can sense potential hazards and then conduct calibration and validation.

## 2. Related Work

Perception of dangerous situation (PDS) includes two aspects: sensing a dangerous state and recognizing a dangerous situation. It refers to perceiving and understanding various dangerous components in the environment within a given time and space [1]. Crundall D. et al. [2] defined danger perception as the ability to quickly perceive danger. They defined that hazard perception ability is a driver’s ability to sense impending danger. Drivers with strong danger perception abilities can effectively detect impending dangers and react immediately to avoid accidents. Accurate and effective measurement of a driver’s dangerous situation perception ability is of great significance for measuring and evaluating a driver’s safe driving level [3,4]. Many countries have incorporated hazard perception tests into driver’s license examinations. Bruce C. et al. [5] studied the role of the Occupational Therapy Risk Propensity Test (OT-RiPT) in risk perception testing and verified its reliability. Long S. et al. [6] developed a dangerous situation prediction test, “What’s Next”, for Chinese drivers and evaluated its reliability and validity. Glen F.C. et al. [7] simulated and designed a danger perception test for drivers under different levels of visual impairment. This is utilized to assess the danger perception ability of drivers with visual impairment and whether they are capable of driving on the road. Malone S. et al. [8] compared the effects of traditional test tasks, language tasks, and motor tasks on the measurement of drivers’ hazard perception ability. They also pointed out that three different types of tasks should be considered in the danger perception test to better assess the driver’s danger perception ability. Sepulveda J. A. et al. [9] studied the relationship between the motion sensitivity of drivers’ central and peripheral vision and hazard perception tests. The results indicate that the driver’s central and peripheral visual motion sensitivity is related to their reaction time in hazard perception tests, while the driver’s binocular visual acuity is not related to their reaction time in hazard perception tests. Meir A. et al. [10] believed that the ability to perceive and detect danger is an ability that increases with age and experience. They then studied the danger perception abilities of children and experienced adults when crossing the road. Mcinerney K. et al. [11] studied the neuropsychological factors related to the danger perception ability of older drivers. In their study, they demonstrated that there is a significant correlation between hazard perception ability and visual-spatial/structural skills, processing speed, memory, and executive function in healthy older drivers. Sun L. et al. [12] developed a danger perception test for cyclists to assess their ability to perceive hazards during cycling. Jing B. et al. [13] proposed an automatic assessment network for drivers’ danger perception ability in highway scenarios under icy and snowy conditions.

Currently, potential risks or hidden dangers have also been addressed in a few studies on danger perception. Wei T. et al. [14] designed three types of explicit and implicit dangerous events based on the UC-win/Road driving simulation software. The driver’s ability to perceive danger and behavioral characteristics of different explicit and implicit events was studied. This was used to guide the design of the driver’s danger perception ability test, so as to measure and improve the driver’s danger perception ability. Qian Y.B. et al. [15] proposed a collision avoidance trajectory planning algorithm, which enables autonomous vehicles to actively avoid blind spots and remain clear of areas where the field of vision is blocked, thereby reducing the potential collision risk caused by visual field obstruction. He X.L. et al. [16] proposed a Potential Collision Avoidance Difficulty model (PCAD) to calculate the risks around a vehicle in real time and explain its underlying mechanisms. The acceleration and velocity parameters required for the vehicle to avoid a collision in real time were calculated. Gao Z.H. et al. [17] considered using multidimensional factors of uncertainty, which include driver behavior, sensor perception, motion prediction models, and road structure, to estimate the collision risk between the current vehicle and surrounding vehicles, thereby proactively avoiding or mitigating potential collisions. Waymo recently released the Waymo Open Dataset for End-to-End (WOD-E2E) dataset and proposed the Rate Feedback Score (RFS) as an evaluation metric [18]. It focuses on end-to-end autonomous driving research in long-tail scenarios and designs an evaluation system aligned with human preferences. Furthermore, it explicitly states that evaluating the performance of an autonomous driving system should not only focus on whether it replicates the actual driving route of a human driver but also on whether its behavior is as reasonable, safe, and natural as that of an experienced driver. However, although their work mentions the need to learn from experienced drivers, they do not point out the characteristics of experienced drivers, nor do they propose any methods to screen out such experienced drivers.

## 3. Methodology

### 3.1. Experiment

#### 3.1.1. Experimental Participants

Generally, novice drivers often lack the ability to perceive dangerous omens or exhibit poor performance in perceiving a dangerous omen. Therefore, the experimental personnel in this study are selected from experienced “veteran drivers” with a large driving mileage (greater than 100,000 km). The participants for the vehicle driving experiment are recruited from the full-time drivers of the National Intelligent Connected Vehicle Innovation Center—Vehicle-Road-Cloud Integrated Test Demonstration Base, while the participants for the virtual driving test are selected through social recruitment. Each participant can receive a cash reward upon successful completion of the experiment, with a reward of 100 CNY per person for the vehicle driving experiment and 50 CNY per person for the virtual driving experiment. A total of 134 drivers participated in the experiment, which included 56 in the vehicle driving experiment and 78 participants in the virtual driving experiment. The test drivers range in age from 28 to 50 years old, with an average age of 39.78 years. In addition, three experimental assistants participated in the experiment.

#### 3.1.2. Experimental Equipment and Scene

**Figure 1:**
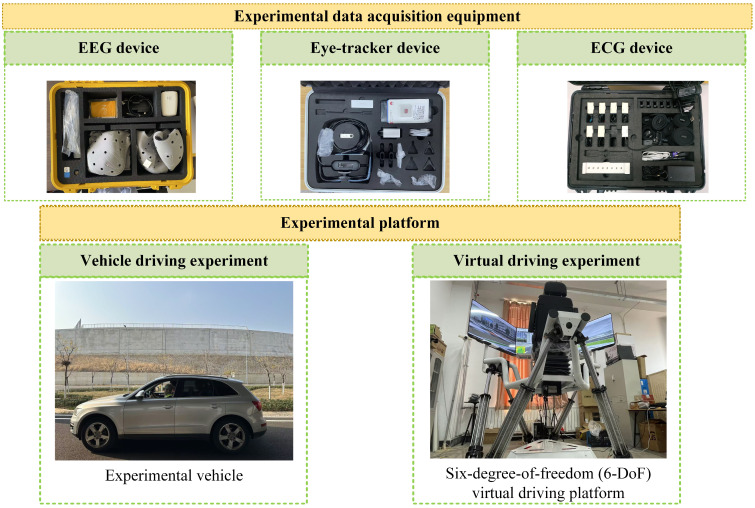
Experimental equipment.

**Figure 2:**
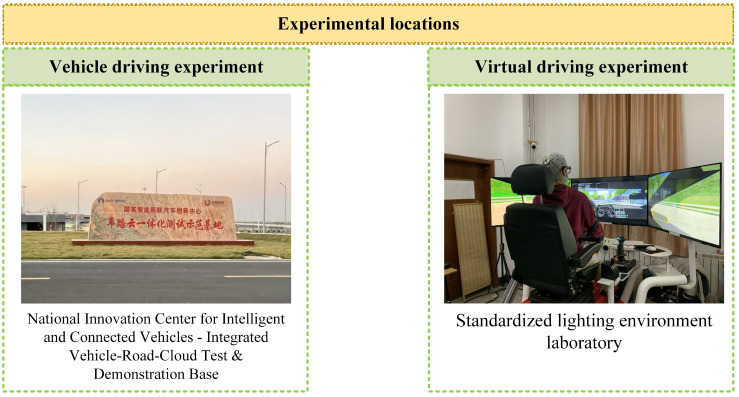
Experimental locations. The Chinese name in the left figure is "National Innovation Center for Intelligent and Connected Vehicles–Integrated Vehicle-Road-Cloud Test & Demonstration Base”.

**Figure 3:**
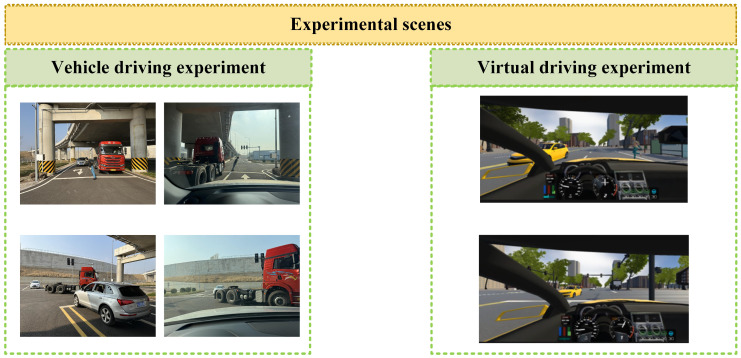
Experimental scenes.

Both vehicle driving and virtual driving experiments are included in the experiments. Data such as EEG, electrocardiogram (ECG), and eye movement are collected. The vehicle driving experiments are conducted at the National Intelligent Connected Vehicle Innovation Center—Vehicle-Road-Cloud Integrated Testing and Demonstration Base in Zhaoyuan City, Shandong Province, China, while the virtual driving experiment is carried out in a laboratory with standard lighting conditions. Two typical ghost-peeking scenarios are designed into the experimental scene. The experimental equipment and setup are shown in Figure 1, Figure 2 and Figure 3. The EEG device used is from Yingfu Technology (Shanghai, China), model NIC2. The eye tracker used is from Seven Invensun (Beijing, China), model aSee Studio Glasses. The ECG equipment used is from Jinfa Technology (Shanghai, China), model ErgoLab.

#### 3.1.3. Experiment Design

The experiments conducted in this study are divided into vehicle and virtual driving experiments. The experimental procedures for both are roughly the same. However, for safety reasons, during the vehicle driving experiment, the experimental assistant in the passenger seat uses the secondary brake to brake the vehicle at any time in case of an emergency. Participants are unaware of the research details before the experiment. They are simply required to drive the car normally and safely within the predetermined experimental scenario to complete the driving task. The specific experimental procedure is as follows:

(1) Before the experiment begins, the experimental assistants confirm that all experimental equipment is usable. Experimental assistants help the participants put on experimental equipment such as eye trackers, electroencephalograms, electrocardiograms, and electrodermatology devices. And they set up video recording equipment inside and outside the vehicle. The in-vehicle experimental video can use the eye tracker’s recording function to record the driver’s first-person perspective information. The external video equipment uses a drone to record from a top–down perspective of the car. After confirming that everything is in order, preparations for the experiment begin.

(2) Participants enter either the experimental vehicle or the virtual driving experimental platform. Experimental assistants activate each device. Subjects are required to complete driving tasks in the two constructed experimental scenarios according to a predetermined driving route.

(3) Experimental assistants record the entire experimental process using pre-set video recording equipment and collect EEG, ECG, and eye movement data of the test drivers during the experiment.

(4) After each driving task is completed, the experimental assistant and the driver watch the video playback of the driving task together. The assistants ask and record whether the driver perceived a dangerous omen at the time when the danger occurred.

(5) After all participants complete their driving tasks, all experimental data are saved. The experimental equipment is cleaned up. The experiment is terminated.

### 3.2. Local Variable Point Statistics of EEG Data

In this study, the local analysis method in variable point statistical analysis is used to analyze the EEG data of drivers. Local analysis [19,20] is a practical and powerful tool in the field of change point detection, especially in modern data flow and real-time monitoring applications. Its core lies in “seeing the big picture from the local,” which achieves rapid early warning of process changes by continuously scanning recent data. In this study, the sliding window method, commonly used in local analysis, is utilized to determine whether the driver could perceive dangerous omens and the specific time point at which the dangerous omen is perceived. The specific method is as follows:

Let the brainwave power time series be $X=\{x_{1},x_{2},\cdots ,x_{n}\}$. The core idea of the sliding window detection algorithm is to identify change points by comparing the statistical characteristics of data in adjacent windows. Define the position of the detection window $W_{t}$ at time point t:

(1) $$W_{t}=\{x_{t-w+1},x_{t},x_{t+1},\cdots ,x_{t+w-1}\}$$

The detection window is divided into two sub-windows of equal length. The left window is defined as $L_{t}=x_{t-w+1},\ldots ,x_{t}$ and the right window as $R_{t}=x_{t+1},\ldots ,x_{t+w}$, where w is the half-length of the window.

For each time point t, calculate the mean difference statistic between the left and right windows:

(2) $$T(t)=\frac{{\bar{R}}_{t}-{\bar{L}}_{t}}{\hat{\sigma }\cdot \sqrt{\frac{2}{w}}}$$

${\bar{L}}_{t}=\frac{1}{w}\sum i=t-w+1^{t}x_{i}$ is the mean of the left window. $\bar{R}t=\frac{1}{w}\sum i=t+1^{t+w}x_{i}$ is the mean of the right window. $\hat{\sigma }t=\sqrt{\frac{\sum i=t-w+1^{t}{\left(x_{i}-\bar{L}t\right)}^{2}+\sum i=t+1^{t+w}{\left(x_{i}-{\bar{R}}_{t}\right)}^{2}}{2w-2}}$ is the pooled standard deviation.

When the statistical test statistic exceeds a predefined threshold, a change point is detected. The change point detection criteria are as follows:

(3) $$\left|T(t)\right|>\tau$$

Alpha wave data is used as an example in this study; the sequence of characteristic values $X_{1},X_{2},\ldots ,X_{n}$ of the collected EEG alpha waves at different time points is denoted as $X_{1},X_{2},\ldots ,X_{n}$. The specific statistical procedure for detecting local change points is as follows: t=1,2,…,n.

(1) Construct a local comparison statistic $Y_{i}$ Set a local window half-width. For each candidate point i(d+1≤i≤n−d), calculate the local comparison statistic $Y_{i}$ using the following formula:

(4) $$Y_{i}=(\sum_{j=i}^{i+d-1}X_{j})-(\sum_{j=i-d}^{i-1}X_{j})$$

By constructing and calculating the local comparison statistic $Y_{i}$, we compare the sum of EEG features of the current window [i,i+d−1] with that of the previous window [i,i+d−1]. If the features near point i are stable, then $Y_{i}$ is close to zero. If a sudden change occurs at point i, then $Y_{i}$ is significantly non-zero.

(2) Estimate the location of the change point

Calculate all the maximum values $\left|Y_{i}\right|$ and their corresponding positions using the following formula:

(5) $$\left|Y_{i^{*}}\right|=\underset{d+1\leq i\leq n-d}{\max}\left|Y_{i}\right|$$

(6) $$\hat{m}=i^{*}$$

$\hat{m}$ is the initial estimate of the change point location.

(3) Hypothesis testing is used to determine whether there is a significant change point.

Let the null hypothesis $H_{0}$ be: there is no change point at this location (i.e., no significant change in EEG features). Construct the test statistic:

(7) $$W=\left|Y_{i^{*}}\right|$$

The formula for calculating the critical value C is as follows:

(8) $$C=\sigma \cdot \sqrt{2d}\cdot A_{n}(x_{\alpha })$$

σ is the standard deviation of $X_{t}$. If W>C is true, then we reject $H_{0}$ and conclude that a significant change point exists at $\hat{m}$.

(4) Jump size estimation

If a change point is confirmed, the average jump amplitude $\hat{\theta }$ of the α wave can be calculated using the following formula:

(9) $$\hat{\theta }=\frac{1}{n-\hat{m}+1}\sum_{t=\hat{m}}^{n}X_{t}-\frac{1}{\hat{m}-1}\sum_{t=1}^{\hat{m}-1}X_{t}$$

For other waveforms, the process of local change point detection is the same as for α waves. But the input feature sequence distribution needs to be adjusted based on the actual collected data, resulting in different local change point detection results. In this study, the detailed parameter settings of the sliding window for EEG data are shown in Table 1. Specifically, the window lengths (3 s and 4 s) are selected based on both the typical temporal scale of hazardous omen perception (3–6 s) and the sampling rate requirement (250 Hz). The detection threshold (μ + 3σ) corresponds to a strict significance level of *p* < 0.0013 under the normality assumption, ensuring that detected change points reflect genuine neural responses with a very low false positive rate. The significance level of *p* < 0.1 reported in Table 1 does not refer to the initial detection threshold, but rather to the screening criterion used in the hypothesis testing stage for the local change point statistic—this relatively lenient threshold is adopted to retain sensitivity to weak but genuine signals while strictly controlling overall false alarms. The minimum duration threshold of 0.5 s effectively excludes transient physiological artifacts such as blinks and myoelectric activity (typically <200 ms) and is consistent with the cognitive time window required for the transition from subconscious processing to conscious awareness of a dangerous omen.

**Figure 4:**
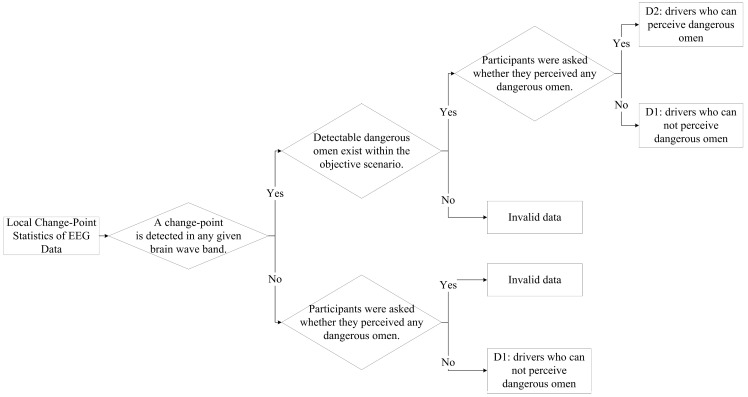
The specific process for determining whether a driver can perceive dangerous omens.

For each participating driver, local change point analysis needs to be performed on their brainwave data, including α, β, θ, δ, and γ waves. To determine whether a driver can perceive a dangerous omen, a combined strategy of detecting multiple EEG changes is employed in this study, along with video playback of the experiment and proactive questioning of the driver, which can jointly assess the dangerous omen recognition results and the timing of their perception. The specific process is as follows: Sliding window detection is applied to each electroencephalogram (EEG) dataset. When no change points are detected in any of the EEG datasets, the driver is actively questioned, and those who perceived no dangerous omens during the experiment are classified as D1. When a change point is detected in any type of electroencephalogram (EEG) data, combined with video playback and direct questioning of the driver, drivers who could not perceive the impending dangerous omen are still classified as D1, while those who could perceive dangerous omens are classified as D2, considering this change point as the time point of dangerous omen perception. Figure 4 shows a schematic diagram of the specific process for determining whether a driver can perceive impending dangerous omens.

### 3.3. Driver Screening Model Based on the Structure Equation Model

The structural equation model is a type of linear structural relationship model capable of handling latent variables. It is widely used in research fields such as psychology, education, human factors engineering, and behavioral science. In this model, latent variables can be measured indirectly through certain observed indicators. Advantages of this structural equation model lie in its quantitative study of interactions among multiple variables. Multiple dependent variables can be considered and handled simultaneously. Measurement errors in both independent and dependent variables are allowed. Factor structures and factor relationships can be estimated concurrently. Complex models in which an indicator belongs to multiple factors can be analyzed. Additionally, the overall fit of the model can be evaluated. In this structural equation model, variables include observed variables and latent variables. Observed variables refer to those that can be directly obtained. Latent variables refer to those that cannot be directly obtained but can be derived from observed variables.

**Figure 5:**
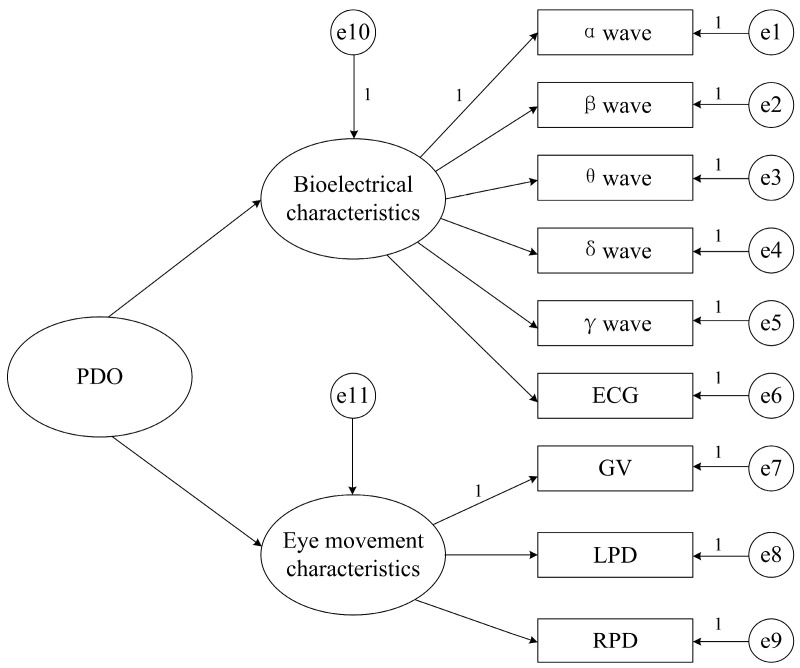
Structural path diagram for calculating the risk perception ability of car drivers.

Our study collects two types of physiological characteristics from drivers, namely bioelectrical signals (EEG α waves, EEG β waves, EEG θ waves, EEG δ waves, EEG γ waves, and ECG) and eye movement characteristics (GV, LPD, and RPD). Therefore, when calculating the risk perception ability of drivers, both the bioelectrical signals (EEG α waves, EEG β waves, EEG θ waves, EEG δ waves, EEG γ waves, and ECG) and the eye movement characteristics (GV, LPD, and RPD) should be taken into account. Finally, based on the statistical test results, irrelevant features can be filtered out by setting their weights to zero. In this study, bioelectrical features and eye movement features are treated as latent variables. The directly collected driver data for each type of feature are utilized as observed indicators. An initial model for calculating drivers’ dangerous omen perception ability is thus established. The structural path diagram of the model is shown in Figure 5, where e1, e2, …, e9 represent the measurement errors of the different observed variables, and e10 and e11 represent the residuals of the latent variables. In simple terms, the structural equation model in this study can be understood as a “Driving Dangerous Omen Perception Ability Calculator”. This calculator takes nine physiological indicators (five EEG frequency bands, ECG, and three eye movement metrics) as its input and produces a score as output—the driver’s hazardous omen perception ability value.

## 4. Results

### 4.1. Statistical Analysis of Physiological Data

In this study, electroencephalogram (EEG), electrocardiogram (ECG), and eye-tracking data are obtained from 134 participants during vehicle and virtual driving. To avoid pseudoreplication, all statistical analyses were conducted at the driver level. For each participant, the median value of each feature was used as its representative measure. That is, for each feature, the data from each driver were aggregated into a single observation, and these observations were independent across different drivers. Examples of the data are shown in Table 2. The electroencephalogram (EEG) data includes α waves, β waves, θ waves, δ waves, and γ waves. Eye movement data includes gaze velocity (GV), left pupil diameter (LPD), and right pupil diameter (RPD).

#### 4.1.1. EEG

Table 3 shows the descriptive statistics of the electroencephalogram (EEG) data collected from the two types of drivers through experiments.

For the analysis of data from two samples, the independent samples *t*-test is generally considered. However, this method requires that the data satisfy the conditions of normal distribution and homogeneity of variances. With driver type as the factor variable, and α, β, θ, δ, and γ as the dependent variables, Kolmogorov–Smirnov tests and tests for homogeneity of variance are performed. The condition of homogeneity of variance is not met, and the results indicate that the data do not follow a normal distribution. Therefore, non-parametric tests are required for the EEG data of different driver types. Since the two sets of parameters under analysis are independent samples, the Mann–Whitney U test is selected in this study. The results are shown in Table 4.

**Figure 6:**
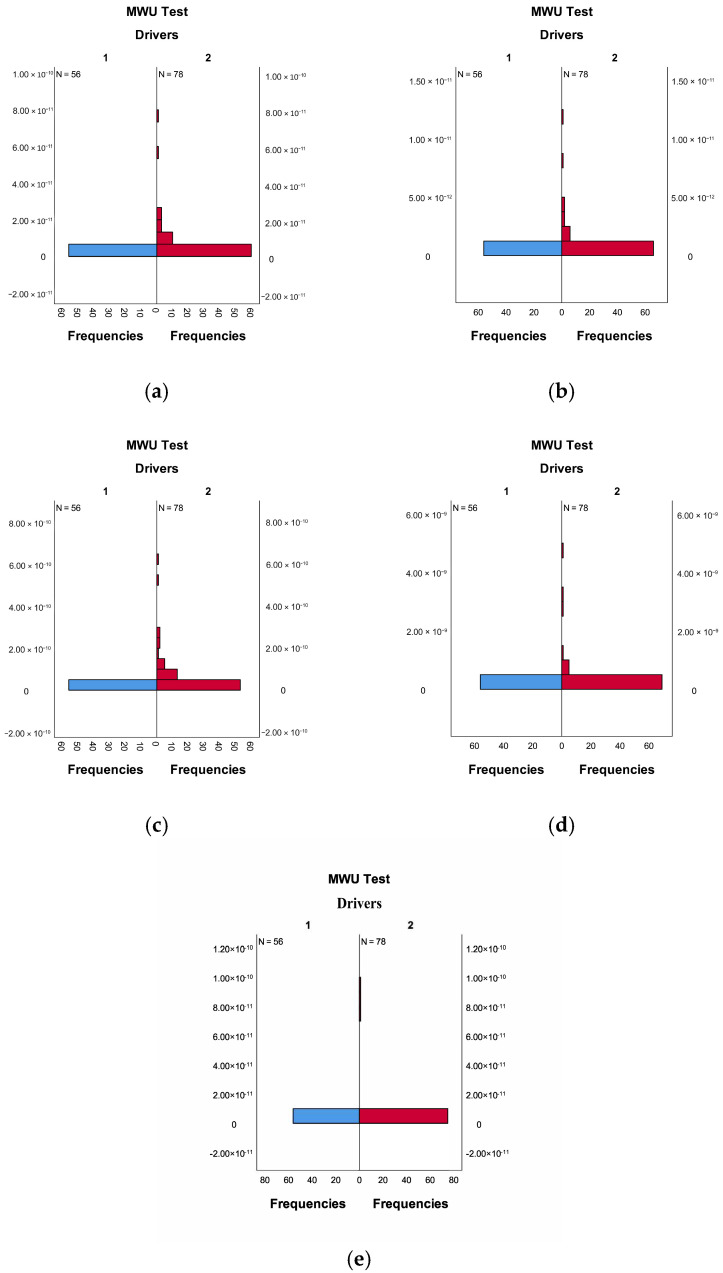
Distributions of α, β, θ, δ, and γ wave data for different types of drivers. (**a**) α wave; (**b**) β wave; (**c**) θ wave; (**d**) δ wave; and (**e**) γ wave. In the figure, 1 and 2 represent driver types D1 and D2, respectively.

In the Mann–Whitney U test, the null hypothesis for this study is set as follows. Different driver types have identical distributions of α, β, θ, δ, and γ. As shown in Table 4, the significance levels for α (*p* < 0.001), β (*p* = 0.014), θ (*p* < 0.001), δ (*p* < 0.001), and γ (*p* = 0.048) in different driver types are all less than 0.05. The null hypothesis is therefore rejected. This indicates that the distributions of α, β, θ, δ, and γ differ significantly between driver types. It is thus demonstrated that significant differences exist in the EEG α, β, θ, δ, and γ data between D1 and D2 driver types. Figure 6 presents the distribution of α, β, θ, δ, and γ data for different driver types. As shown in Figure 6, a distinct difference in the distributions of EEG α, β, θ, δ, and γ data between the two driver types can also be visually observed.

#### 4.1.2. ECG

Table 5 shows the descriptive statistics of the electrocardiogram data collected from the two types of drivers through experiments.

With driver type as the factor variable and ECG data as the dependent variable, Kolmogorov–Smirnov tests and homogeneity of variance tests are performed on the data. The results indicate that these data do not follow a normal distribution. Therefore, non-parametric tests are required for the ECG data of different driver types. The results obtained after the Mann–Whitney U test are shown in Table 6.

**Figure 7:**
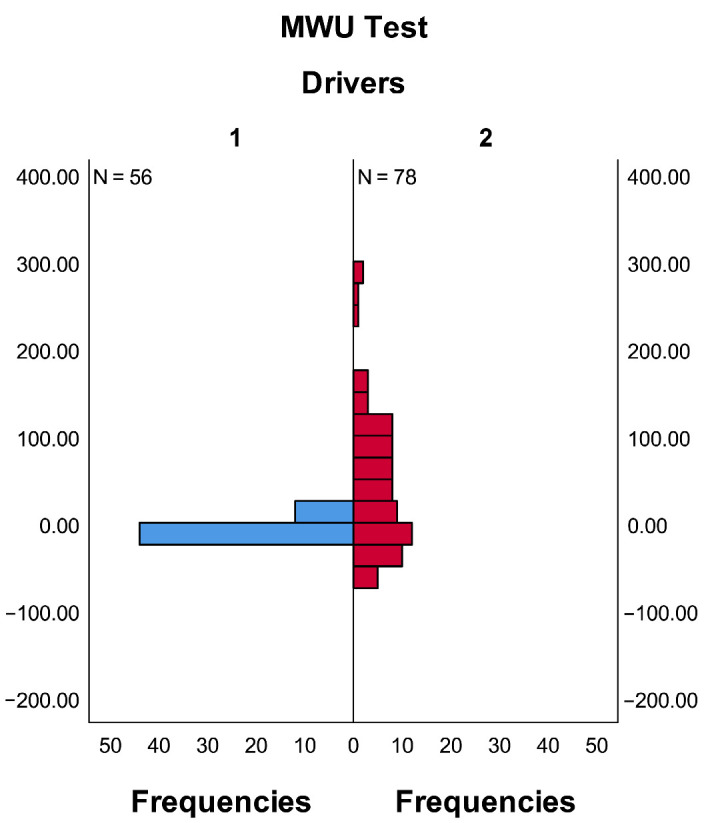
Distribution of ECG data for different types of drivers. In the figure, 1 and 2 represent driver types D1 and D2, respectively.

In the Mann–Whitney U test, the null hypothesis for this study is defined as follows. The distribution of ECG data is identical across different driver types. As shown in Table 6, the significance level of ECG data for different driver types (*p* = 0.001) is less than 0.05. The null hypothesis is therefore rejected, indicating that the distribution of ECG data differs significantly between driver types. In other words, a statistically significant difference exists in ECG data between D1 and D2 driver types. The distribution of ECG data for different driver types is presented in Figure 7, from which the distinct difference in the distribution of ECG data between the two driver types can also be visually observed.

#### 4.1.3. Eye Movement

The descriptive statistical results of eye movement characteristics during the dangerous omen perception process for the two driver types, collected through the experiment, are summarized in Table 7.

With driver type as the factor variable and GV, LPD, and RPD as the dependent variables, Kolmogorov–Smirnov tests and homogeneity of variance tests are performed on the data. The test results indicate that these data do not satisfy the normal distribution assumption. Therefore, non-parametric tests are required for the eye movement data of different driver types. The results obtained after the Mann–Whitney U test are presented in Table 8.

**Figure 8:**
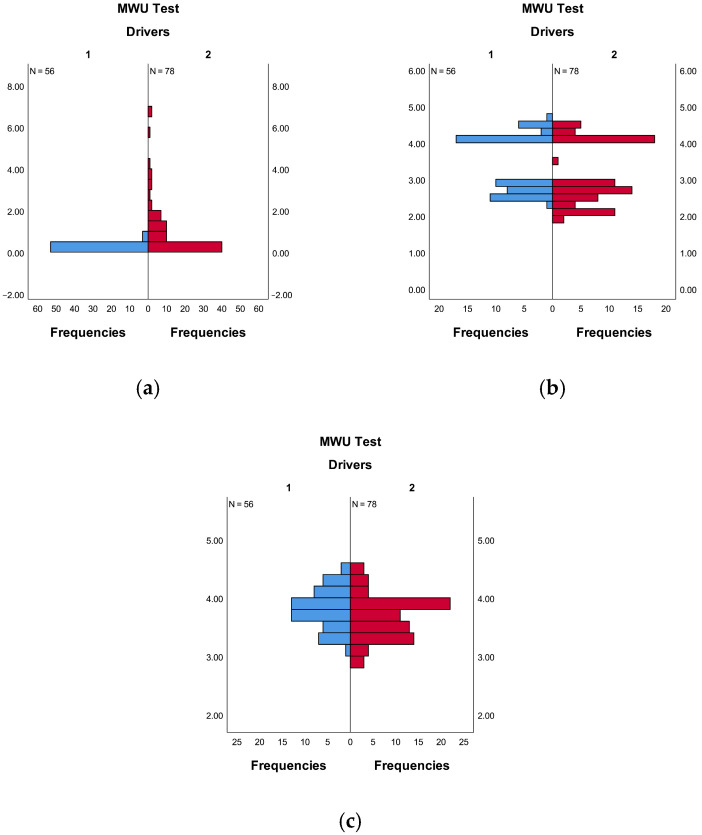
Distributions of GV, LPD, and RPD data for different types of drivers. (**a**) GV; (**b**) LPD; (**c**) RPD. In the figure, 1 and 2 represent driver types D1 and D2, respectively.

In the Mann–Whitney U test, the null hypothesis for this study is set as follows. The distributions of GV, LPD, and RPD data are identical across different driver types. As shown in Table 8, the significance levels for GV (*p* < 0.001), LPD (*p* = 0.021), and RPD (*p* = 0.032) in different driver types are all below 0.05. The null hypothesis is therefore rejected, indicating that the distributions of GV, LPD, and RPD data differ significantly between driver types. That is, statistically significant differences exist in GV, LPD, and RPD data between D1 and D2 driver types. The distributions of GV, LPD, and RPD data for different driver types are presented in Figure 8, from which a clear visual distinction in the distribution of eye movement data (GV, LPD, and RPD) between the two driver types can also be observed.

The statistical analysis of EEG, ECG, and eye movement data from D1 and D2 driver types indicates that distinct EEG, ECG, and eye movement characteristics are exhibited by drivers capable of dangerous omen perception and those incapable of it prior to accidents. The significant difference between the two driver types in perception of dangerous omen is therefore demonstrated. It is confirmed that some drivers can anticipate and recognize warning signs before accidents occur, which means that the perception ability of a dangerous omen is possessed by these drivers. This perception ability of a dangerous omen of drivers is intended to be utilized in so-called brain-inspired intelligence. The ability is to be applied to autonomous vehicles, so that potentially dangerous omens can be predicted and identified by them. Consequently, risks can be assessed earlier, and measures can be taken in advance. The probability of accidents is thus reduced.

### 4.2. Calibration and Validation of Driver Screening Models

The entire cohort of 134 drivers was first stratified based on their true labels (D1/D2), and 20% of individuals were randomly selected from each stratum to form an independent test set (D1: 11, D2: 16, total 27). This test set was completely sealed off during the entire process of model training, parameter estimation, and cross-validation, and was used only once for final evaluation. The data from the remaining 107 drivers were used for model training. In this study, k-fold cross-validation (k = 5) was employed for model training and validation. Specifically, the 107 drivers were randomly divided into five mutually exclusive subsets, with the proportion of D1 and D2 drivers kept consistent across each subset. For each fold k (k = 1, 2, 3, 4, 5), the k-th subset was used as the validation set, while the remaining four subsets were combined as the training set for model training and validation. The performance metrics from the five validation folds were averaged to obtain the cross-validation results. After model training and validation, the previously untouched independent test set of 27 drivers was used for model testing. The final fully standardized output results of the structural equation model are presented in Table 9.

Here, $\lambda_{ij}$ is the factor loading coefficient of each observed variable on its corresponding latent variable, indicating the importance of the observed variable relative to the latent variable. It can thus be regarded as the weight of the observed variable. $\rho_{i}$ is the path coefficient, which reflects the importance of each latent variable in relation to dangerous omen perception ability. It can therefore be considered as the weight of the latent variable. The formula for calculating the driver’s dangerous omen perception ability (Perception of Dangerous Omen, PDO) is derived as shown in the following equation.

(10) $$PDO=\rho_{1}\sum_{i,j=1}^{6}\lambda_{1j}E_{i}+\rho_{2}\sum_{i,j=1}^{3}\lambda_{2j}G_{i}$$

Here, $E_{i}$ is the driver’s bioelectrical characteristics. $G_{i}$ corresponds to the eye movement characteristics. Based on the *PDO* values associated with different driver types (D1, D2, and D3), three intervals are established. Each interval corresponds to the dangerous omen perception ability of a specific driver type. The results are shown in Table 10. The results indicate that the value range of dangerous omen perception ability for drivers capable of perceiving a dangerous omen is [0.3125, 1].

The standardized data are input into the initially constructed model for calculating drivers’ perception ability of dangerous omens. After fitting, the calculation results of drivers’ perception ability of dangerous omens are obtained. Drivers with a PDO value greater than or equal to 0.3125 are classified as those capable of perceiving dangerous omens, while those with a value less than 0.3125 are classified as incapable. A driver screening model is thus established, which utilizes the calculated perception ability of dangerous omens to screen drivers. The goodness-of-fit evaluation parameters of the constructed model are presented in Table 11.

As shown in Table 11, the RMSEA is less than 0.08. The CFI, NFI, TLI, and GFI indices, which represent the overall goodness-of-fit of the model, all exceed 0.9. This indicates that the model has a high degree of fit with the observed data. The model is validated using the k-fold cross-validation method, with the results shown in Table 12.

**Figure 9:**
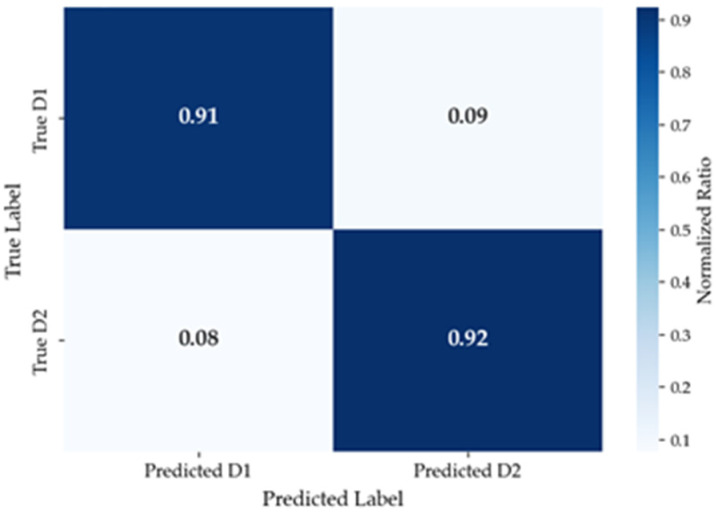
Confusion matrix of k-fold validation results when k = 5.

The confusion matrix of the model validation results under this setting is shown in Figure 9.

The model is tested using the experimental data reserved in this study. By inputting the electroencephalogram (EEG), eye movement, and electrocardiogram (ECG) data of drivers of different categories into the driver screening model, the model test results are obtained as shown in Table 13.

**Figure 10:**
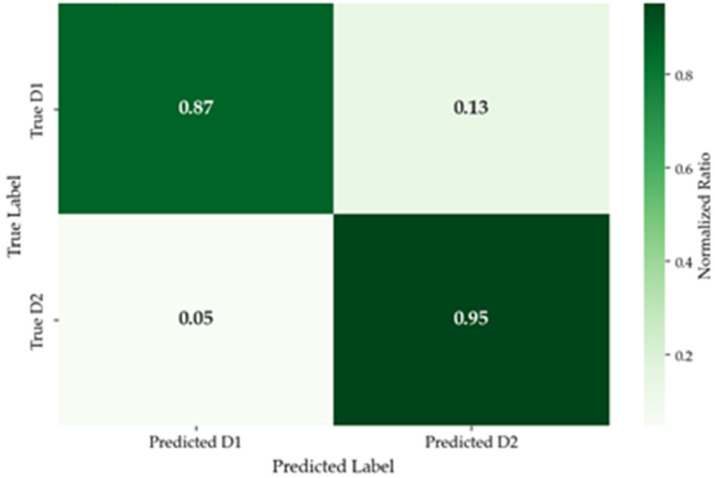
Confusion matrix of test results.

As can be seen from the results in Table 13, the driver screening model constructed in this study demonstrates excellent performance. The confusion matrix of the model test results is shown in Figure 10.

To validate the effectiveness of the proposed model, this study designed baseline model comparison and ablation experiments. All experiments were conducted using the exact same training, validation, and test datasets as those used for the SEM model (i.e., data from 107 drivers for training/validation, data from 27 drivers for testing, with 5-fold cross-validation). Three representative benchmark classifiers were selected: Logistic Regression (LR), Support Vector Machine (SVM), and Random Forest (RF). In addition, to quantify the contribution of each feature modality, four feature subsets were constructed under the same structural equation modeling framework: EEG-only, eye movement-only, ECG-only, and eye movement + ECG. The latent variable structures of the ablation models were adjusted accordingly, while all other modeling procedures remained consistent with the full SEM model. Table 14 summarizes the results of the baseline model comparison and ablation experiments. The results in the table are all the results obtained on the test set.

As shown in Table 14, the proposed SEM model outperformed all baseline models in terms of classification performance. Within the SEM framework, using EEG features alone achieved an accuracy of 80.1%, demonstrating that EEG characteristics are strongly associated with dangerous omen perception ability. Models using only ECG (77.3%) or only eye movement features (77.9%) exhibited relatively low performance; however, the eye movement + ECG fusion model (85.2%) showed substantial improvement, indicating that drivers’ dangerous omen perception ability can still be distinguished even without EEG features.

## 5. Discussions and Limitations

### 5.1. Discussions

When assessing whether drivers can perceive dangerous omens, this study adopts combining change-point analysis of drivers’ electroencephalogram (EEG) data with experimental video playback and active questioning of the drivers. Prior to the experiment, participants were not informed of any research-related details. They were only instructed to drive safely and complete the route from the starting point to the destination. After each driver completes the experiment, change-point analysis is immediately performed on their EEG data. Based on these results, the experimental assistant and participant jointly review the driving recording, and the participant is asked whether they had perceived a dangerous omen at the time corresponding to each valid change point. This can avoid the subjective bias that could arise if drivers knew the purpose and content of the study in advance. Moreover, conducting the video review and questioning immediately after the experiment can reduce the influence of memory decay or hindsight bias.

Our study employs statistical analysis methods such as the Kolmogorov–Smirnov test and non-parametric tests to determine whether D1 and D2 drivers exhibit different physiological characteristics during the perception of dangerous omens. According to the results presented in Table 4, Table 6 and Table 8, the two driver types show different distributions in α, β, θ, δ, and γ brainwaves, ECG, GV, LPD, and RPD data. That is, significant differences exist in their EEG, ECG, and eye movement features, indicating that drivers’ ability to perceive dangerous omens is significantly correlated with their EEG, ECG, and eye movement characteristics. Thus, different types of drivers possess different levels of ability to perceive dangerous omens, and this ability can be quantified and distinguished using physiological data. According to statistical analysis results, this study constructs a driver screening model based on a structural equation model. During model calibration, even features with relatively low weights (e.g., certain waveform indices) were retained in the final model. This is because the statistical results indicate that all these features are significantly correlated with drivers’ ability to perceive dangerous omens. Thus, their influence should not be ignored regardless of weight magnitude. Furthermore, cross-validation is introduced, and the results further demonstrate the validity and precision of the constructed model. Using this model, the ability to perceive dangerous omens of drivers can be calculated, and drivers capable of perceiving dangerous omens can be identified accordingly, thereby achieving driver screening.

### 5.2. Limitations

EEG signals are used both in the labeling procedure (as a localizer) and in the subsequent modeling (as predictive features). This is indeed a limitation and an area for further improvement. However, this circularity does not invalidate our core findings. As shown in Figure 7 and Figure 8, eye movement and ECG data exhibit clearly distinguishable distributions between D1 and D2 drivers—particularly ECG. The ablation study results also demonstrate that even if EEG features are completely excluded, a model built solely on eye movement and ECG data can still effectively distinguish between the two driver groups. Our decision to retain EEG features in the final model is based on two considerations: (1) Not all EEG data are used in the labeling process (only one of the EEG wave types—α, β, θ, δ, or γ—may have been used for change point detection). In the candidate point selection step, a time point is flagged as a candidate if any one of the multiple EEG bands shows a change point. The final perception moment is then determined by combining the objective hazard timing and the driver’s verbal confirmation. Thus, the majority of EEG data are never involved in label definition. (2) EEG provides unique theoretical and predictive values. EEG directly reflects cortical information processing and offers incremental predictive power beyond peripheral physiological signals. Incorporating EEG makes the model more complete and neurocognitively grounded. In future studies, EEG-independent labeling methods will be further explored, and online reporting paradigms that do not require disclosing the experimental purpose in advance will be developed.

In the preliminary exploratory phase of this study, attempts were made to inform drivers beforehand that they needed to perceive dangerous omens during the experiment for collecting their perception timing through real-time questioning or button-press feedback. However, this approach led to a problem: some drivers with low or no ability to perceive dangerous omens remained highly tense during the experiment, constantly wondering whether a dangerous omen had appeared. As a result, they often reported perceiving the dangerous omen before it actually occurred, generating a large amount of false alarm data. Therefore, our method is further refined: participants were not informed of any research-related information in advance (and those who had already learned about the study were excluded from further participation). Instead, change-point analysis of EEG data combined with post-experiment video review and immediate questioning is used to determine whether a driver could perceive dangerous omens and to identify the exact moment of perception. Since drivers remained unaware of the research objectives until the review and questioning stage, this actually reduces the influence of subjective bias. However, this approach does not completely eliminate the influence of subjective cognition. Some drivers have a vague awareness of dangerous omens, and they may subconsciously perceive a potentially dangerous omen but do not form a clear conscious recognition of it. This can result in change points in their EEG data, while their responses during questioning remain ambiguous or uncertain. Such cases did not occur in the experiments and were classified as D1-type drivers (those who were unable to perceive dangerous omens). This classification is based on the objective of this study: to identify drivers who can genuinely perceive dangerous omens, so that their capability of perceiving dangerous omens can be referenced and applied to autonomous vehicles, thereby enhancing the proactive safety of such vehicles. In other words, our aim is to identify D2-type drivers and ensure that they are truly capable of perceiving dangerous omens, as only the abilities of such drivers hold meaningful reference value for autonomous driving systems. Therefore, this limitation does not substantially affect the experimental outcomes of this study. In future research, more appropriate methods should be explored, and fully objective data collection approaches should be attempted to reduce the impact of drivers’ subjective bias.

The experiments were conducted only under controlled scenarios (two typical ghost-peeking situations), covering both simulated and real-vehicle driving. While this design ensures internal validity, it has not yet been validated or generalized to more diverse driving contexts with higher ecological validity. In future research, validation can be extended to more complex road environments and different weather conditions.

The sample size of this study (N = 134) is moderate, and the independent test set (27 drivers) is relatively small. To enhance the robustness of the results, we employed 5-fold cross-validation. However, the uncertainty regarding the model’s generalization performance still needs to be further addressed. In future studies, the experimental scale can be expanded by collecting data from more drivers under richer scenarios to increase the sample size.

Although the proposed screening model is theoretically grounded in structural equation modeling, it currently remains at an exploratory stage. The factor structures and path coefficients were estimated based on the current dataset and have not yet been tested on external samples. Future research should validate the model on independent populations and, where conditions permit, employ confirmatory factor analysis or cross-dataset validation to further strengthen the evidence.

Despite the above limitations, this study establishes a methodological foundation for quantifying drivers’ hazardous omen perception ability and provides empirical evidence and validation. The findings offer valuable insights and inspiration for the development of brain-inspired intelligence in autonomous driving.

## 6. Conclusions

Drivers with a high level of agility and sensitivity are able to extract features of a dangerous omen during the process from initial abnormality to emerging danger. Potential collision risks can thus be predicted by them. Accidents are better avoided, and driving safety is effectively ensured. The concept of “brain” in brain-inspired intelligence includes the cognitive functions of such drivers. Therefore, the screening of drivers capable of perceiving dangerous omens is regarded as an important prerequisite. This ability can then be transferred to the field of autonomous vehicles. A driver screening method based on dangerous omen perception tests is proposed in this study. Drivers are distinguished based on whether the dangerous omen can be perceived by them. Differences in bioelectrical and eye movement characteristics during the dangerous omen perception process are also analyzed. It is confirmed that some drivers are indeed able to anticipate and recognize potentially dangerous omens. On this basis, a structural equation model is applied. The dangerous omen perception ability of drivers is calculated. A driver screening model is constructed, calibrated, and validated accordingly. The physiological, psychological, and behavioral characteristics of drivers who can perceive a dangerous omen are thoroughly examined in this study. A screening method for such drivers is also proposed. In further research, objective scenarios related to a dangerous omen can be explored based on driver screening results. These scenarios refer to the driving scenes observed from the first-person perspective when dangerous omens are perceived by such drivers. Driving behaviors adopted in these scenarios can also be analyzed. From these studies, vehicles can learn from the perception and response behaviors of drivers in accident-prone situations. In this study, exploratory insights are offered for enhancing the brain-inspired intelligence of vehicles, methodological inspiration is provided for the development of end-to-end autonomous driving, and conceptual contributions are made to the improvement of road traffic safety. Future research should focus on validating these findings in larger, more diverse cohorts and under real-world driving conditions.

## Figures and Tables

**Table 1:** Sliding window parameters for EEG data in the local analysis method.

	Window Length	Number of Sampling Points	Sampling Frequency	Threshold	Minimum Duration	Significance Level
α	4 s	1000	250 Hz	μ + 3σ (*p* < 0.1)	0.5 s	0.042 (*p* < 0.1)
β	4 s	1000	250 Hz	μ + 3σ (*p* < 0.1)	0.5 s	0.087 (*p* < 0.1)
θ	3 s	750	250 Hz	μ + 3σ (*p* < 0.1)	0.5 s	0.035 (*p* < 0.1)
δ	3 s	750	250 Hz	μ + 3σ (*p* < 0.1)	0.5 s	0.061 (*p* < 0.1)
γ	3 s	750	250 Hz	μ + 3σ (*p* < 0.1)	0.5 s	0.029 (*p* < 0.1)

**Table 2:** Examples of experimental data (partial).

	Number	α (μV^2^/Hz)	β (μV^2^/Hz)	θ (μV^2^/Hz)	δ (μV^2^/Hz)	γ (μV^2^/Hz)	GV (px/ms)	LPD (mm)	RPD (mm)	ECG (μV)
Vehicle driving experiment	1	2.08 × 10^−13^	1.10 × 10^−13^	3.34 × 10^−13^	1.34 × 10^−12^	6.24 × 10^−14^	0.019	4.040	3.948	−4.770
	2	1.41 × 10^−12^	5.22 × 10^−13^	4.96 × 10^−12^	5.39 × 10^−11^	2.59 × 10^−13^	0.103	4.001	3.886	−1.626
	3	8.02 × 10^−13^	2.43 × 10^−13^	7.82 × 10^−12^	2.58 × 10^−11^	1.35 × 10^−13^	0.242	4.040	3.851	−0.588
	4	4.56 × 10^−13^	1.28 × 10^−13^	3.33 × 10^−12^	2.13 × 10^−11^	5.51 × 10^−14^	0.069	4.184	4.178	−1.516
	5	5.98 × 10^−13^	2.02 × 10^−13^	1.82 × 10^−12^	6.76 × 10^−12^	5.86 × 10^−14^	0.075	4.518	4.555	1.405
	6	6.81 × 10^−13^	1.77 × 10^−13^	1.73 × 10^−12^	8.95 × 10^−12^	7.80 × 10^−14^	0.465	4.155	4.103	0.069
	7	1.72 × 10^−12^	2.12 × 10^−13^	1.24 × 10^−11^	5.01 × 10^−11^	1.02 × 10^−13^	0.141	4.167	4.098	−1.823
	8	3.11 × 10^−13^	1.53 × 10^−13^	5.17 × 10^−13^	1.47 × 10^−12^	6.16 × 10^−14^	0.163	4.180	4.212	0.153
	…	…	…	…	…	…	…	…	…	…
	55	2.50 × 10^−12^	3.26 × 10^−13^	2.49 × 10^−11^	1.03 × 10^−10^	8.20 × 10^−14^	0.446	2.408	3.309	−1.276
	56	2.68 × 10^−12^	2.30 × 10^−13^	4.27 × 10^−11^	1.14 × 10^−10^	7.29 × 10^−14^	0.120	2.465	3.226	−1.891
Virtual driving experiment	1	5.12 × 10^−12^	1.73 × 10^−13^	7.06 × 10^−11^	1.86 × 10^−10^	7.37 × 10^−14^	0.056	2.778	3.384	149.589
	2	5.09 × 10^−12^	1.98 × 10^−13^	5.57 × 10^−11^	1.71 × 10^−10^	5.11 × 10^−14^	0.421	2.810	3.412	168.390
	3	2.71 × 10^−12^	1.02 × 10^−13^	4.65 × 10^−11^	1.55 × 10^−10^	8.72 × 10^−14^	0.794	2.889	3.511	25.432
	4	1.02 × 10^−12^	2.14 × 10^−13^	7.49 × 10^−12^	2.76 × 10^−11^	8.14 × 10^−14^	0.045	2.918	3.526	8.138
	5	7.05 × 10^−13^	1.11 × 10^−13^	1.41 × 10^−12^	4.98 × 10^−12^	6.59 × 10^−14^	0.352	2.725	3.510	−7.894
	6	3.06 × 10^−13^	1.28 × 10^−13^	8.00 × 10^−13^	2.44 × 10^−12^	6.81 × 10^−14^	0.460	2.717	3.377	−23.929
	7	5.80 × 10^−13^	2.01 × 10^−13^	2.91 × 10^−12^	2.08 × 10^−11^	5.79 × 10^−14^	3.688	2.814	3.604	139.963
	8	6.36 × 10^−13^	2.45 × 10^−13^	2.02 × 10^−12^	6.59 × 10^−12^	8.97 × 10^−14^	1.099	4.169	3.950	−55.997
	…	…	…	…	…	…	…	…	…	…
	77	7.01 × 10^−12^	1.78 × 10^−12^	1.25 × 10^−10^	7.59 × 10^−10^	3.61 × 10^−13^	1.493	4.531	4.221	124.398
	78	2.17 × 10^−11^	4.92 × 10^−12^	6.40 × 10^−10^	2.71 × 10^−9^	8.74 × 10^−13^	0.555	4.599	4.334	56.977

**Table 3:** Descriptive statistics of electroencephalogram (EEG) data for different types of drivers.

EEG Features	Driver Type	Total *N*	Average Value (μV^2^/Hz)	Standard Deviation	95% Confidence Interval of the Mean		Minimum Value (μV^2^/Hz)	Maximum Value (μV^2^/Hz)
					Lower Limit	Upper Limit		
α	D1	56	5.812 × 10^−13^	4.869 × 10^−13^	4.507 × 10^−13^	7.115 × 10^−13^	1.554 × 10^−13^	2.678 × 10^−12^
	D2	78	5.769 × 10^−12^	1.119 × 10^−11^	3.245 × 10^−12^	8.292 × 10^−12^	8.491 × 10^−14^	7.391 × 10^−11^
β	D1	56	1.709 × 10^−13^	7.518 × 10^−14^	1.507 × 10^−13^	1.910 × 10^−13^	9.126 × 10^−14^	5.222 × 10^−13^
	D2	78	7.752 × 10^−13^	1.792 × 10^−12^	3.714 × 10^−13^	1.179 × 10^−12^	7.085 × 10^−14^	1.203 × 10^−11^
θ	D1	56	3.091 × 10^−12^	6.576 × 10^−12^	1.330 × 10^−12^	4.852 × 10^−12^	3.335 × 10^−13^	4.267 × 10^−11^
	D2	78	6.005 × 10^−11^	2.182 × 10^−11^	3.579 × 10^−11^	8.431 × 10^−11^	2.127 × 10^−13^	6.398 × 10^−10^
δ	D1	56	1.530 × 10^−11^	2.412 × 10^−11^	8.836 × 10^−12^	2.176 × 10^−11^	1.170 × 10^−12^	1.142 × 10^−10^
	D2	78	2.818 × 10^−10^	6.893 × 10^−10^	1.263 × 10^−10^	4.372 × 10^−10^	9.553 × 10^−13^	4.559 × 10^−9^
γ	D1	56	8.173 × 10^−14^	3.421 × 10^−14^	7.257 × 10^−14^	9.089 × 10^−14^	3.800 × 10^−14^	2.586 × 10^−13^
	D2	78	4.709 × 10^−13^	1.653 × 10^−11^	9.827 × 10^−13^	8.835 × 10^−12^	3.923 × 10^−14^	9.140 × 10^−11^

**Table 4:** Results of the Mann–Whitney U test on EEG data from different types of drivers.

	Driver Type	M (P25, P75)	Two-Sample Rank Sum Test	
			Z	*p*
α	D1	4.582 × 10^−13^ (3.238 × 10^−13^, 6.320 × 10^−13^)	5.939	0.000
	D2	2.135 × 10^−12^ (2.221 × 10^−13^, 6.333 × 10^−12^)		
β	D1	1.547 × 10^−13^ (1.179 × 10^−13^, 1.981 × 10^−13^)	2.455	0.014
	D2	1.999 × 10^−13^ (1.237 × 10^−13^, 4.891 × 10^−13^)		
θ	D1	1.198 × 10^−12^ (6.804 × 10^−13^, 2.745 × 10^−12^)	6.471	0.000
	D2	2.182 × 10^−11^ (2.879 × 10^−12^, 7.308 × 10^−11^)		
δ	D1	4.279 × 10^−12^ (2.099 × 10^−12^, 1.747 × 10^−11^)	6.288	0.000
	D2	1.087 × 10^−10^ (1.336 × 10^−11^, 2.179 × 10^−10^)		
γ	D1	7.206 × 10^−14^ (6.432 × 10^−14^, 8.661 × 10^−14^)	1.980	0.048
	D2	8.596 × 10^−14^ (5.802 × 10^−13^, 9.118 × 10^−13^)		

**Table 5:** Descriptive statistics of electrocardiogram data for different types of drivers.

Driver Type	Total *N*	Average Value (μV^2^/Hz)	Standard Deviation	95% Confidence Interval of the Mean		Minimum Value (μV^2^/Hz)	Maximum Value (μV^2^/Hz)
				Lower Limit	Upper Limit		
D1	56	−2.377	4.436	−3.541	−1.214	−13.437	9.155
D2	78	47.384	80.659	29.198	65.570	−74.096	289.998

**Table 6:** Results of the Mann–Whitney U test on ECG data of different types of drivers.

	Driver Type	M (P25, P75)	Two-Sample Rank Sum Test	
			Z	*p*
ECG	D1	−1.853 (−4.984, −0.134)	3.280	0.001
	D2	36.538 (−13.184, 97.288)		

**Table 7:** Descriptive statistics of eye movement characteristics for different types of drivers.

Eye Movement Feature	Driver Type	Total *N*	Average Value (μV^2^/Hz)	Standard Deviation	95% Confidence Interval of the Mean		Minimum Value (μV^2^/Hz)	Maximum Value (μV^2^/Hz)
					Lower Limit	Upper Limit		
GV	D1	56	0.163	0.172	0.117	0.209	0.018	0.938
	D2	78	1.088	1.426	0.767	1.410	0.018	6.637
LPD	D1	56	3.413	0.807	3.197	3.629	2.350	4.758
	D2	78	3.108	0.845	2.918	3.299	1.865	4.599
RPD	D1	56	3.813	0.336	3.723	3.903	3.166	4.555
	D2	78	3.663	0.373	3.579	3.747	2.831	4.494

Note: The average, minimum, and maximum values for gaze velocity in the table are in px/ms, and the average, minimum, and maximum values for left and right pupil diameters are in mm.

**Table 8:** Results of the Mann–Whitney U test on eye movement data of different types of drivers.

	Driver Type	M (P25, P75)	Two-Sample Rank Sum Test	
			Z	*p*
GV	D1	0.108 (0.064, 0.217)	6.277	0.000
	D2	0.470 (0.213, 1.495)		
LPD	D1	2.976 (2.623, 4.165)	−2.307	0.021
	D2	2.801 (2.482, 4.057)		
RPD	D1	3.810 (3.572, 4.060)	−2.145	0.032
	D2	3.716 (3.382, 3895)		

**Table 9:** Standardized weights of the structural equation model.

Latent Variable	Observed Variables	Coefficient	Standardized Factor Loadings
Bioelectrical Characteristics	α	$\lambda_{11}$	0.452
	β	$\lambda_{12}$	0.123
	θ	$\lambda_{13}$	0.289
	δ	$\lambda_{14}$	0.511
	γ	$\lambda_{15}$	−0.028
	ECG	$\lambda_{16}$	0.015
Eye Movement Characteristics	GV	$\lambda_{21}$	0.008
	LPD	$\lambda_{22}$	−0.012
	RPD	$\lambda_{23}$	0.035
Perception Ability of Dangerous Omen	Bioelectrical characteristics	$\rho_{1}$	1.000
	Eye Movement Characteristics	$\rho_{2}$	0.068

**Table 10:** Hazard Perception Abilities of Different Types of Drivers.

Driver Type	D1	D2
*PDO*	[0.000, 0.3125)	[0.3125, 1]

**Table 11:** Model Fitting Evaluation Parameters.

Evaluation Metrics	Numerical Value
Chi-squared/Degrees of freedom	4.273
Root mean square error of approximation (RMSEA)	0.054
Comparative fit index (CFI)	0.974
Normed Fit Index (NFI)	0.965
Tucker–Lewis index (TLI)	0.958
Goodness of Fit Index (GFI)	0.986

**Table 12:** Model Validation Results.

Value of k	Accuracy	Precision	Recall	F1-Score
1	0.936	0.938	0.930	0.934
2	0.942	0.944	0.939	0.940
3	0.949	0.951	0.945	0.948
4	0.945	0.947	0.941	0.944
5	0.958	0.959	0.956	0.957

**Table 13:** Model Test Results.

Evaluation Metrics	Value (95%CI)
Accuracy	0.917 (0.857–0.977)
Precision	0.929 (0.871–0.977)
Recall	0.867 (0.795–0.939)
F1-Score	0.897 (0.840–0.954)

**Table 14:** Baseline model comparison and ablation experiments results.

Model	Accuracy (%)	Precision (%)	Recall (%)	F1-Score (%)
LR	78.5 ± 2.1	78.0 ± 2.3	79.0 ± 2.0	78.5 ± 2.2
SVM	79.2 ± 1.9	78.8 ± 2.1	79.5 ± 1.8	79.1 ± 1.9
RF	81.0 ± 1.7	80.5 ± 1.9	81.3 ± 1.6	80.9 ± 1.8
SEM with only EEG	80.1 ± 1.8	79.5 ± 2.0	80.8 ± 1.7	80.1 ± 1.9
SEM with only Eyemovement	77.9 ± 2.0	77.1 ± 2.2	78.6 ± 1.9	77.8 ± 2.1
SEM with only ECG	77.3 ± 2.2	76.6 ± 2.4	78.0 ± 2.1	77.3 ± 2.3
SEM with ECG and Eyemovement	85.2 ± 1.5	84.6 ± 1.6	85.3 ± 1.4	84.9 ± 1.5
SEM with all characteristics	91.7 ± 1.2	92.9 ± 1.1	86.7 ± 1.8	89.7 ± 1.3

## Data Availability

The data presented in this study are available on request from the corresponding author. The data are not publicly available due to privacy.

## Abbreviations

The following abbreviations are used in this manuscript:

PDS	Perception of Dangerous Situation
OT-RiPT	Occupational Therapy Risk Propensity Test
PCAD	Potential Collision Avoidance Difficulty
WOD-E2E	Waymo Open Dataset for End-to-End
RFS	Rate Feedback Score
K-S	Kolmogorov–Smirnov
MWU	Mann–Whitney U
GV	Gaze Velocity
LPD	Left Pupil Diameter
RPD	Right Pupil Diameter
Fp1	Frontal Pole 1
Fpz	Frontal Pole Zero
Fz	Frontal Zero
FC1	Frontal Central 1
FC2	Frontal Central 2
F4	Frontal 4
PDO	Perception of Dangerous Omen
EEG	Electroencephalogram
ECG	Electrocardiogram
